# Quantification of species composition in grass-clover swards using RGB and multispectral UAV imagery and machine learning

**DOI:** 10.3389/fpls.2024.1414181

**Published:** 2024-06-19

**Authors:** Joanna Pranga, Irene Borra-Serrano, Paul Quataert, Tom De Swaef, Thijs Vanden Nest, Koen Willekens, Greet Ruysschaert, Ivan A. Janssens, Isabel Roldán-Ruiz, Peter Lootens

**Affiliations:** ^1^ Plant Sciences Unit, Flanders Research Institute for Agriculture, Fisheries and Food (ILVO), Melle, Belgium; ^2^ Research Group Plants and Ecosystems (PLECO), Department of Biology, University of Antwerp, Wilrijk, Belgium; ^3^ Institute of Agricultural Sciences, Spanish National Research Council (ICA-CSIC), Madrid, Spain

**Keywords:** OBIA, drone, supervised classification, pasture, *Lolium*, *Trifolium*, high-throughput field phenotyping

## Abstract

**Introduction:**

Growing grass-legume mixtures for forage production improves both yield productivity and nutritional quality, while also benefiting the environment by promoting species biodiversity and enhancing soil fertility (through nitrogen fixation). Consequently, assessing legume proportions in grass-legume mixed swards is essential for breeding and cultivation. This study introduces an approach for automated classification and mapping of species in mixed grass-clover swards using object-based image analysis (OBIA).

**Methods:**

The OBIA procedure was established for both RGB and ten band multispectral (MS) images capturedby an unmanned aerial vehicle (UAV). The workflow integrated structural (canopy heights) and spectral variables (bands, vegetation indices) along with a machine learning algorithm (Random Forest) to perform image segmentation and classification. Spatial k-fold cross-validation was employed to assess accuracy.

**Results and discussion:**

Results demonstrated good performance, achieving an overall accuracy of approximately 70%, for both RGB and MS-based imagery, with grass and clover classes yielding similar F1 scores, exceeding 0.7 values. The effectiveness of the OBIA procedure and classification was examined by analyzing correlations between predicted clover fractions and dry matter yield (DMY) proportions. This quantification revealed a positive and strong relationship, with R2 values exceeding 0.8 for RGB and MS-based classification outcomes. This indicates the potential of estimating (relative) clover coverage, which could assist breeders but also farmers in a precision agriculture context.

## 1 Introduction

In European temperate grasslands, grasses and legumes are frequently cultivated in mixtures (Skovsen et al., 2017), with the main legume species being white clover (*Trifolium repens* L.) and red clover (*Trifolium pratense* L.) (Rognli et al., 2021). The main reason is that grass-clover mixtures can perform better than each of the component species in monoculture, in terms of yield and/or nutritional quality (Nyfeler et al., 2011; Fujiwara et al., 2022). Owing to their ability to capture atmospheric nitrogen through a symbiotic relationship with soil bacteria, legumes generally are rich in protein content (Sun et al., 2021). A key advantage of incorporating legumes into grassland mixtures stems from the fact that nitrogen fixed by legumes can be transferred to neighboring non-legume plants (e.g. grass) (Pirhofer-Walzl et al., n.d; Thilakarathna et al., 2016). Establishing grass-legume swards by combining species with varying aboveground characteristics and root architecture (species niche complementarity) offers other benefits, including an increase in biodiversity (Rochon et al., 2004) and other positive consequences for the environment (Lüscher et al., 2014). Such mixtures have demonstrated enhanced resistance to weed invasion when compared to monocultures (Deak et al., 2007). Introducing legumes can also help restore nitrogen levels in the soil, thus reducing the need for fertilizer application (Khatiwada et al., 2020).

A forage with a high clover content can offer a high feed quality (Sun et al., 2021) and can increase fodder intake by livestock (Dewhurst et al., 2009; Mortensen et al., 2017). Consequently, the objective of forage production is to maintain a predetermined proportion of the legume component (Skovsen et al., 2017), while maximizing the annual dry matter yield. However, maintaining such well-balanced grass-legume mixtures over time is challenging (Lüscher et al., 2014) due to various environmental factors (e.g. soil fertility, temperature, soil moisture content, species and cultivar choice) that play a crucial role in the growth and persistence of clover in mixed swards (Botha, 2009), as well as management factors (e.g. cutting regime and nitrogen fertilization) which affect the competition dynamics between grass and clover (Sun et al., 2021). For instance, the grass-clover ratio can be regulated by manipulating the amount of nitrogen fertilizer applied (Skovsen et al., 2017). With ample soil nitrogen availability (high fertilization), the faster-growing grass will outcompete clover. On the contrary, with limited soil-available nitrogen, clover will dominate the sward (Skovsen et al., 2017). An optimal quantity and quality of forage is reached when the clover fraction ranges from 30% to 50% in total dry matter (Botha, 2009). A higher percentage of clover usually leads to a notable decrease in the yield (Khatiwada et al., 2020). Conversely, if clover content falls below 30%, the nutritional value of the feed decreases (Botha, 2009). Therefore, an accurate follow-up of the clover ratio by farmers is essential for targeted management regarding fertilization or reseeding, and to estimate the nutritional value of the forage (Biewer et al., 2009; Himstedt et al., 2010; Skovsen et al., 2017).

A common method used for estimating the clover fraction in grass-clover swards in the context of breeding and research involves destructive sampling: cutting vegetation subsamples, followed by manual separation of the component species (e.g. grasses, legumes etc.), drying and weighing (Himstedt et al., 2010; Mortensen et al., 2017). Due to its labor-intensive and time-consuming nature, this approach is expensive and difficult to employ in practice (Mortensen et al., 2017). A non-destructive and simpler method consists of the visual assessment of species coverage and their composition, in the same way as farmers do. Estimating the clover ratio visually comes with challenges. For instance, to achieve 30% of clover, the pasture must visually display about 50 to 60% of clover coverage because the observable clover content is typically twice its actual amount (AHDB, 2021). In addition, coarse visual inspections are subjective and prone to inaccuracies (Himstedt et al., 2010). They also do not account for spatial variation, both intra-field or across different fields (Skovsen et al., 2017). These limitations have encouraged the advancement of remote sensing (RS) solutions (Li et al., 2021). RS technologies, particularly unmanned aerial vehicles (UAV), offer several advantages in this respect. They are cost-efficient, capable of covering large areas and can capture high-resolution imagery (Deng et al., 2018). These attributes make RS technologies useful for grassland monitoring.

Several RS-based studies that estimate the grass-clover content/ratio in mixed swards through image analysis have been conducted in recent years. The most recent ones focused on the use of deep learning methods for semantic segmentation. For example, Skovsen et al. (2017) trained a fully convolutional network (FCN) to automatically generate a pixel-wise classification of clover, grass, and weeds. Bateman et al. (2020) introduced a new local context network (LC-Net) designed for dense swards and canopies with high occlusion. Sun et al. (2021) utilized not only the FCN architecture but also fine-tuned DeepLab V3+ and SegNet transfer learning methods for clover detection. (Kartal, 2021) compared thirty different segmentation models built with a combination of three deep learning architectures and then randomly initialized encoders. Finally, Fujiwara et al. (2022) employed the fine-tuned GoogLeNet model to estimate legume proportion. Overall, these studies showed that the methods applied are useful to determine the clover fraction in mixed grass-clover swards. A shared characteristic across all these studies is their utilization of RGB imagery acquired with close-range remote sensing; either with a camera setup mounted on a pushcart (Skovsen et al., 2017), on a ground-based platform (Bateman et al., 2020), on a UAV flying at an altitude of 4 m (Fujiwara et al., 2022) or using an Apple iPhone SE camera (Sun et al., 2021). As a result, ultra-high-resolution imagery was captured, revealing fine details of the canopy cover, such as individual grass and clover leaves. Acquiring such high-resolution images and then analyzing them using deep learning techniques comes with challenges, including high computational cost (Justus et al., 2018), extensive manual labeling (Skovsen et al., 2017), and limited field coverage. To address these challenges, in this study we used UAV imagery captured at higher altitudes, thereby facilitating increased spatial coverage up to the field level. Furthermore, we explored the Object-Based Image Analysis (OBIA) approach, to mitigate computational costs and reduce manual labeling efforts. Thus, potentially offering a simplified procedure for the end user while still achieving satisfactory results.

In a traditional pixel-based approach, each pixel is classified separately, and the classification procedure predominantly relies on spectral properties (Blaschke et al., 2014; Zou and Greenberg, 2019). In contrast, OBIA operates at the ‘object level’ (Blaschke, 2010). OBIA segments an image by grouping pixels into non-overlapping objects with a meaningful representation (Blaschke, 2010; Grippa et al., 2017; Hossain and Chen, 2019) and then classifies each object (Blaschke, 2010). This approach was designed primarily to analyze high-spatial resolution imagery by incorporating spectral, shape and textural characteristics as well as spatial patterns (Lang et al., 2009), but is considered an efficient tool for classifying remotely sensed imagery (Lu and He, 2018). Clustering pixels into image objects also aims to overcome the ‘salt and pepper effect’ (Blaschke, 2010). The primary objective of the study presented here was to automatically detect and quantify the percentage of component species in mixed grassland swards using UAV-derived imagery and an OBIA approach. To analyze the effectiveness of the applied method we investigated the relationship between the clover fraction determined using the generated OBIA classification maps and the relative proportion of clover in terms of harvestable dry matter yield determined with destructive methods.

## 2 Materials and methods

### 2.1 Study site

**Figure 1:**
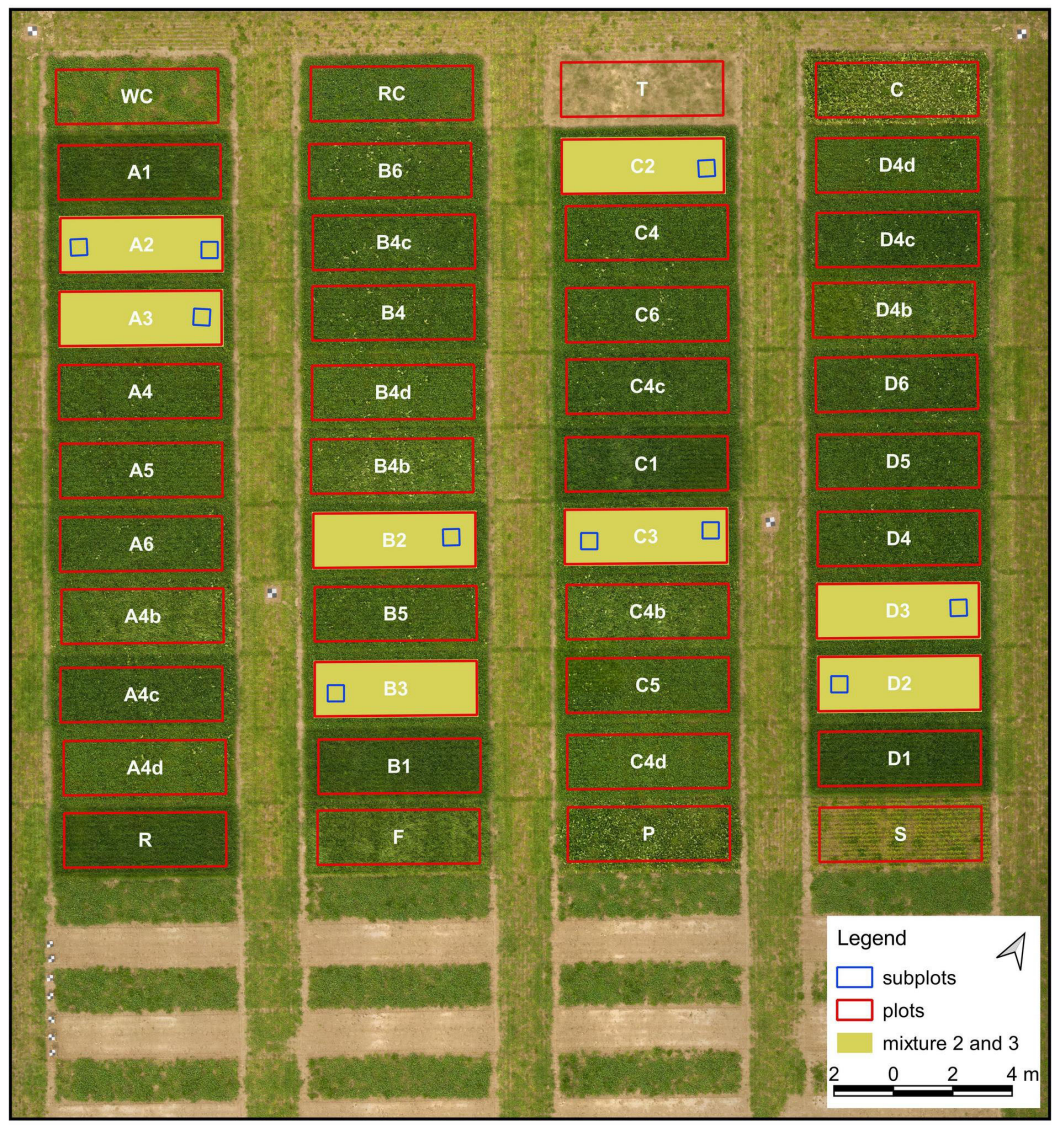
Study site in Merelbeke with a field trial featuring different mixtures of species. Letters A, B, C, and D represent four replicates. Mixture 2: R + WC + RC; mixture 3: R + WC + RC + P. Imagery captured with a UAV-based RGB sensor (collected in May 2021) was set as the base image.

The study was carried out in the municipality of Merelbeke in Belgium (N50°98’, E3°79’; **Figure 1**), using a trial established to test and compare the performance of various species mixtures, when mown frequently (five cuts per year). The field trial was sown in September 2020 with nine different combinations of the following species: perennial ryegrass (*Lolium perenne* L., R), tall fescue (*Festuca arundinacea* Schreb., F), white clover (*Trifolium repens* L., WC), red clover (*Trifolium pratense* L., RC), bird’s-foot trefoil (*Lotus corniculatus* L., T), common sainfoin (*Onobrychis vicifolia* Scop., S), narrowleaf plantain (*Plantago lanceolata* L., P) and common chicory (*Cichorium intybus* L., C). In addition, each species was sown separately in border rows. The mixtures were arranged in a randomized block design with four replicates (A, B, C, D), resulting in a total of 44 plots (2.5 x 6 m).

To address the objectives of this study, only two different combinations (8 plots in total) were selected for further analysis: mixture 2 (grass and clover) and mixture 3 (grass, clover and plantain), marked in yellow in **Figure 1**. Such sward mixtures are commonly grown for forage production in Belgium. The remaining mixtures were not considered in this investigation as this would greatly increase the complexity of the analysis, which was not the aim of this study.

### 2.2 Field sampling and data acquisition

**Figure 2:**
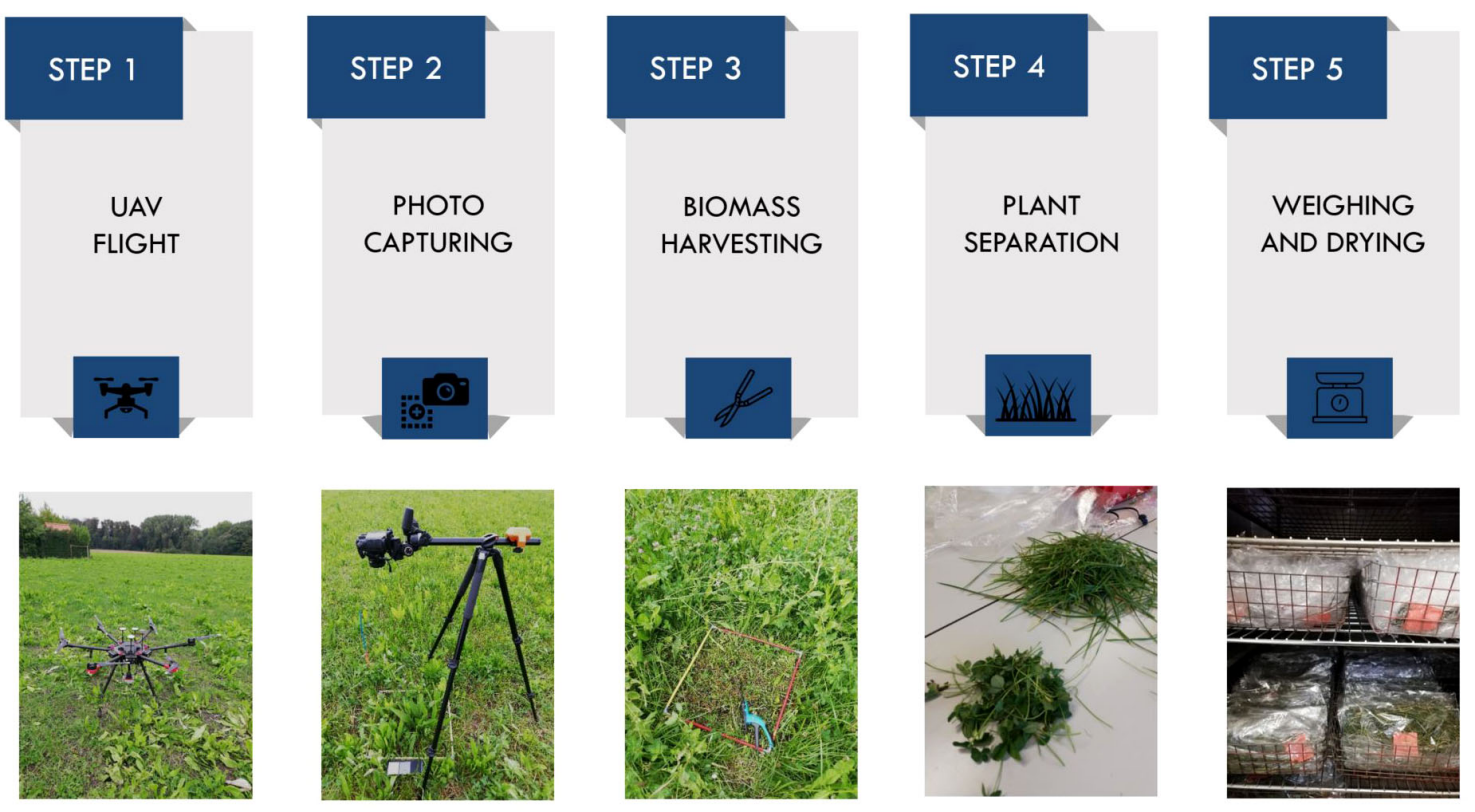
Stages of field sampling and data collection carried out in 2021 at the study site.

For this study, data was collected at the beginning of May (spring cut) of 2021. Field sampling and data acquisition can be divided into five steps (**Figure 2**): (1) UAV-based image collection, (2) reference photo capturing, (3) biomass harvesting, (4) manual separation of component species and (5) weighing and drying of sorted plant material. All the steps are described in detail below.

The first step in the procedure was to perform two drone flights. We utilized a DJI Matrice 600 Pro (DJI, Shenzhen, China) platform and two different sensors: (a) a standard digital RGB camera (α6000, Sony Corporation, Tokyo, Japan) and (b) a multispectral (MS) camera (Dual Camera System, MicaSense, Seattle, USA), with the following ten bands: coastal blue (444 nm), blue (475 nm), green (531 nm), green (560 nm), red (650 nm), red (668 nm), red edge (705 nm), red edge (717 nm), red edge (740 nm), and near-infrared (840 nm). The flight mission was carried out on 6^th^ May around solar noon (2 p.m.). The UAV with each sensor was operated along a pre-defined route and at different altitudes above the ground level. For the RGB camera, the flight altitude was set to 18 m (the lowest possible), as descending further may potentially disrupt the canopy due to the air turbulence generated by the heavy M600 drone propellers. Conversely, with the MicaSense multispectral sensor we followed a recommended minimum flying altitude of 30 meters. Different sensor parameters and flight heights resulted in distinct spatial resolutions, with RGB and MS imagery achieving pixel sizes of 2 mm and 2 cm, respectively. Once the UAV flights were completed, we started a field sampling campaign. Non-destructive and destructive measurements performed in the next steps are not only more time-consuming but also labor-intensive. Therefore, a limited number of subplots were selected within the grass-clover and grass-clover-plantain mixtures (a total of 10 subplots, marked in blue in **Figure 1**). A metal frame (0.55 x 0.55 m in size) and plastic markers were used to define the borders and mark the location of the subplots. First, we captured reference images at ground level (around 1.2 m height) using a tripod and a consumer-grade RGB camera (D90, Nikon Corporation Tokyo, Japan). In the next step, we harvested all aboveground biomass (green vegetation) within the metal frame to a height of 5 cm above soil level. Shortly after the cut, the collected biomass samples were manually separated and sorted into four classes: grass, clover, plantain and weeds. The sorted biomass fractions were oven-dried at 70°C for a minimum of 72 h and weighed. In the last step, the relative proportion (weight-%) of harvestable dry matter yield (DMY) of each fraction related to the total DMY was calculated.

### 2.3 Image processing and image analysis – workflow

**Figure 3:**
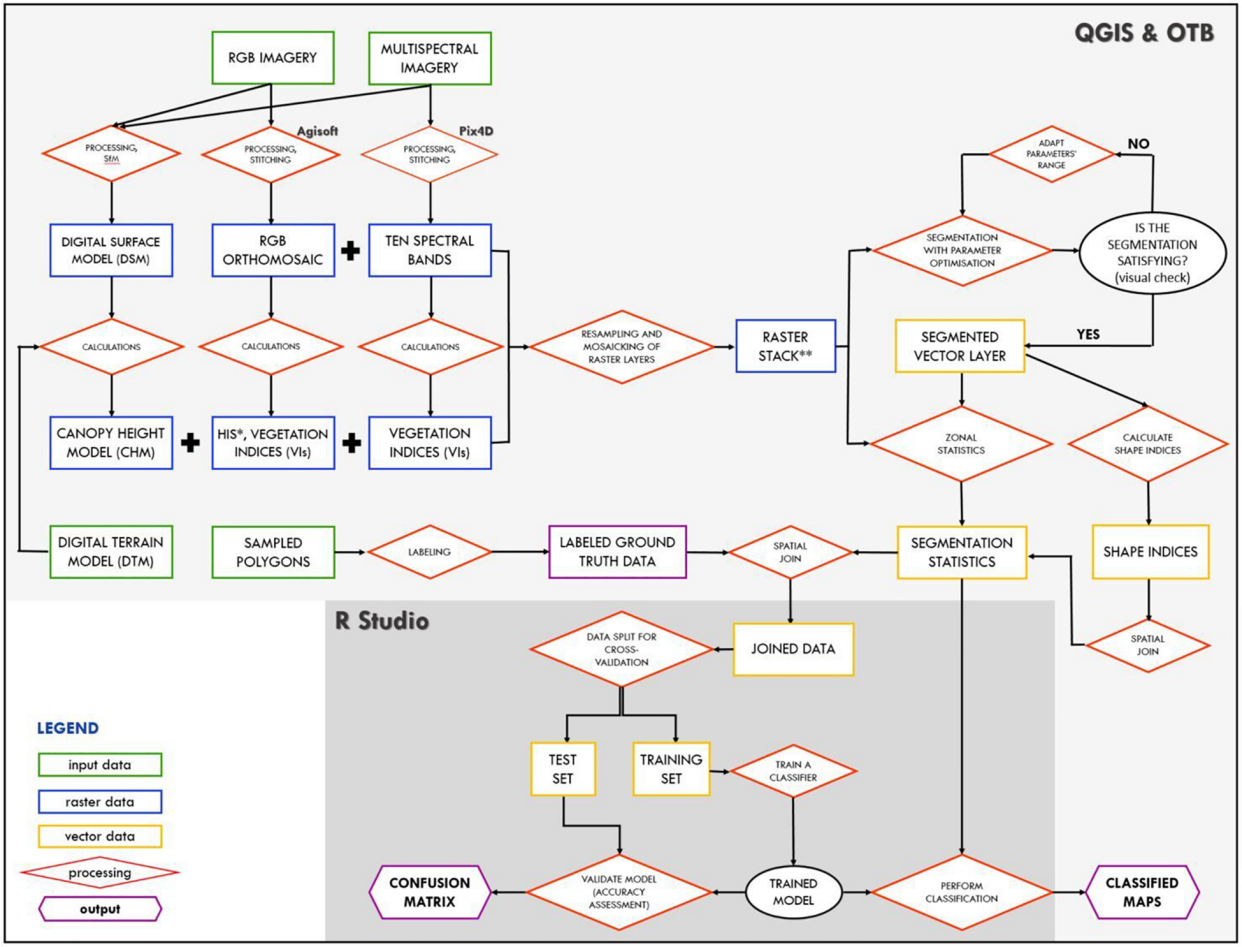
Graphical workflow illustrating the main stages of the processing chain, including image processing, segmentation, image classification and performance assessment (*HIS: Hue, Intensity, and Saturation; ** raster stack of spectral bands and/or vegetation indices).

A graphical workflow (**Figure 3**) represents the key steps of the processing chain, including image processing, calculation of different indices, extraction of information, sampling procedure, segmentation, image classification and performance evaluation, which are described in more detail below.

#### 2.3.1 Image processing

Images acquired with the UAV-based sensors were photogrammetrically processed using Agisoft Metashape Professional (Agisoft LLC, St. Petersburg, Russia) and Pix4DMapper v4.5.6 (Pix4D S.A., Prilly, Switzerland) for the RGB and the multispectral camera, respectively. Both software packages are commonly used for aerial imagery processing and both apply the structure from motion (*SfM*) technique to produce accurate, georeferenced maps and 3D models. The main steps in the image processing procedure for both sensors (and software packages) are similar. They were adopted from our previous study (Pranga et al., 2021), so further details on selected options and parameter settings can be found there. In this study, six Ground Control Points (GCPs), were evenly spread across the field and utilized for precise georeferencing. The geographic coordinates of the GCPs were measured on-site with an RTK GPS (Stonex S10 GNSS, Stonex SRL, Italy).

#### 2.3.2 Image post-processing

As two different sensors were used in this study, the Canopy Height Model (CHM) was computed separately for RGB (CHM_RGB_) and multispectral (CHM_MS_) imagery. The CHM was calculated by subtracting the Digital Surface Model (DSM) and the Digital Terrain Model (DTM) at a pixel level. To compute the DTM, we applied a Triangulated Irregular Network (TIN) interpolation tool. Here, we measured 20 ground points, evenly spread across the study site, with the same RTK GPS.

The RGB orthomosaic created in the previous step was further transformed into hue (H), intensity (I), and saturation (S) color space using the GRASS GIS module with its *i.rgb.his* tool. Similarly, vegetation indices (VIs) were calculated for RGB and MS imagery. Vegetation indices are relatively simple but powerful features that can help in quantitative and qualitative vegetation monitoring and assessment (Xue and Su, 2017). Four VIs were selected for the RGB imagery: (Normalized) Excess Green (ExG), (Normalized) Excess Red (ExR), Excess Green - Excess Red (ExGR), and Normalized Green-Red Difference Index (NGRDI). The MS sensor, with additional data from the red-edge and near-infrared part of the spectrum, provides more possibilities for index calculations. Here, we selected eight indices, comprising Chlorophyll Index Green (Clg), Enhanced Vegetation Index (EVI), Green Atmospherically Resistant Vegetation Index (GARI), Green Normalized Difference Vegetation Index (GNDVI), Modified Chlorophyll Absorption in Reflectance Index (MCARI), and Simple Ratio (SR) with the red-edge band. All vegetation indices were calculated using equations enlisted in the previous study (Pranga et al., 2021). The selection of VIs was based on their general applicability in vegetation monitoring.

A set of further processing steps were applied to the acquired rasters. As digital surface models and maps were generated with different pixel sizes, we started with the raster alignment tool. Here, rasters were resampled to the same cell size and offset in the grid using the nearest neighbor resampling method. The output pixel size was approximately 2 mm and 2 cm for RGB-based and MS-based imagery respectively. All RGB-based rasters (RGB + HIS + CH_RGB_ + VIs) were mosaicked into one raster stack in the next step. The same procedure was repeated for MS-based rasters (10 spectral bands + CH_MS_ + VIs). To perform all these calculations, we used the open-source QGIS 3.22.8 with GRASS 7.8.3. software (QGIS Geographic Information System, QGIS Development Team, Open Source Geospatial Foundation). As the procedure had to be repeated for different sensors, we used the integrated Python console for scripting within the QGIS software (Python version 3.9).

#### 2.3.3 Object-Based Image Analysis

We can divide the OBIA procedure into two main stages: 1) image segmentation and 2) feature extraction and classification.

##### 2.3.3.1 Image segmentation

Image segmentation is the first and key step in OBIA (Lu and He, 2018). During this step, disjoint, spatially continuous and homogenous segments, also known as objects, are generated (Blaschke et al., 2014). The essential objective here is to cluster individual pixels from any raster or image into meaningful objects that should match, as much as possible, the real-world objects (Grippa et al., 2017). A segmentation procedure aims at producing internally homogeneous segments (intra-segment homogeneity) that are distinct from their neighbors (inter-segment heterogeneity) (Espindola et al., 2006). Finding the balance between this intra-segment homogeneity and inter-segment heterogeneity is crucial as it affects the segmentation quality (Grippa et al., 2017; Wijesingha et al., 2020).

In this study, the image segmentation procedure was performed using the open-source project Orfeo ToolBox (OTB) (Grizonnet et al., 2017), which was developed by the Centre National d’Etudes Spatiales (CNES) in France. OTB incorporates many ready-to-use tools for remote sensing (RS) tasks (OTB, 2018), including OBIA-related ones. To create automated processing scripts these tools were applied through the QGIS software with an integrated Python console (Python version 3.9). Image segmentation was performed using the mean-shift algorithm (Michel et al., 2015) option available in the OTB platform, with computation implemented on a tile-wise basis. This way, we could limit memory usage and apply the method to very high-resolution (VHR) imagery (Michel et al., 2015; OTB, 2018). In the mean-shift algorithm, a moving window average is used to group pixels close in the spatial and spectral domain into segments (Comaniciu and Meer, 2002; Hossain and Chen, 2019).

Three key parameters must be set within the OTB mean-shift algorithm: spatial radius (*spatialr*), range radius (*ranger*), and minimum region size (*minsize*) (Teodoro and Araujo, 2016; Varo-Martínez et al., 2017). The first parameter defines the radius of the spatial neighborhood for averaging. The range radius determines the interval in the spectral space (expressed in radiometry units). Thus pixels with a lower range distance than the given parameter value will be grouped into image objects. The last parameter defines the minimum size of a segment (in pixels) to be kept after clustering. Smaller image segments will be merged with the neighboring one that has the closest spectral signature (Teodoro and Araujo, 2016; OTB, 2018; De Luca et al., 2019). The selection of segmentation parameters is a crucial step in the OBIA procedure (Neubert et al., 2008; Grippa et al., 2017), as they control the segmentation quality (Espindola et al., 2006). The most common methods for parameter selection and segmentation evaluation are based on visual and expert interpretations (Zhang et al., 2008; Durgan et al., 2020). Here, a trial-and-error visual approach with a gradual (step-by-step) adaptation of segmentation parameters was implemented. The *ranger* parameter was adapted following the sensor used to obtain the imagery. Pixel values in RGB imagery are recorded as digital numbers (DN), where each band is encoded in the range of 0 to 255. With an MS sensor, a reflectance map is produced, where each pixel indicates the reflectance of the object (values between 0 and 1). The *minsize* parameter was selected based on the geographical context (smallest patch of grass or clover) and spatial resolution of the analyzed imagery (lower resolution corresponds to lower parameter value) (**Table 1**). It has been suggested to set the range of parameter values that will result in under and oversegmented results as extremes (Grippa et al., 2017).

**Table 1:** The list of *spatialr*, *ranger* and *minsize* parameter combinations tested during the segmentation procedure using RGB and MS imagery.

segmentation	RGB (2mm)			MS (2 cm)		
	*spatialr*	*ranger*	*minsize*	*spatialr*	*ranger*	*minsize*
1	10	10	30	5	0.01	2
2	10	10	120	5	0.01	8
3	10	10	200	5	0.01	14
4	10	30	30	5	0.02	2
5	10	30	120	5	0.02	8
6	10	30	200	5	0.02	14
7	30	10	30	15	0.01	2
8	30	10	120	15	0.01	8
9	30	10	200	15	0.01	14
10	30	30	30	15	0.02	2
11	30	30	120	15	0.02	8
12	30	30	200	15	0.02	14

The segmentation procedure was carried out using either RGB-based (RGB + HIS + CH_RGB_) or MS-based (10 spectral bands + CH_MS_) images mosaicked into a raster stack (**Figure 3**; **Table 2**). As a result, a segmented vector layer was generated.

**Table 2:** Variables used for segmentation and/or classification procedure with the description.

CATEGORY	SENSOR	VARIABLES	STATISTIC	USAGE	EXPLANATION
spectral bands	RGB	red, green, blue, hue, intensity, saturation	mean, sd	segmentation, classification	spectral bands of employed sensors
	MS	blue (444 nm), blue (475 nm), green (531 nm), green (560 nm), red (650 nm), red (668 nm), red edge (705 nm), red edge (717 nm), red edge (740 nm), nir (840 nm)	mean, sd	segmentation, classification	
vegetation indices (VI)	RGB	ExG, ExR, EXGR, NGRDI	mean, sd	classification	indices that highlight the difference between spectral properties of plant species; the selection criteria are based on their general applicability in vegetation monitoring
	MS	CIg, EVI, GARI, GNDVI, MCARI, MSAVI, NDVI, SR_717_	mean, sd	classification	
structural features	RGB	CH_RGB_	mean, sd	segmentation, classification	information about the vegetation height above the ground surface
	MS	CH_MS_	mean, sd	segmentation, classification	information about the vegetation height above the ground surface
shape indices (SI)	RGB, MS	A (area), P (perimeter), P/A P/sqrt(A), Sphericity, Shape Index, Dmax, Dmax/A Dmax/sqrt(A)	–	classification	area and perimeter of the object, the inverse of the sphericity, the maximum distance between two polygon vertices, the smoothness of an object border

##### 2.3.3.2 Feature extraction

Before proceeding with image classification, an important step of feature extraction must be completed, as it builds a learning database. In this step, vector layers, generated during the segmentation stage (representing image objects), were used to compute zonal statistics from multiband raster layers. For this purpose, the Zonal Statistics tool from the OTB toolbox was applied. Both spectral and structural features were extracted and used further as predictor variables in the classification process (**Table 2**). The mean and standard deviation statistics were computed for each polygon. Segmented vectors were also used to calculate various shape indices (SI) (**Table 2**), mainly based on area, perimeter and maximum diameter values. To calculate SI, the *Polygon Shape Indices* tool from the SAGA toolbox was applied (Conrad et al., 2015). A recent study by Lam et al. (2021) has shown that selected shape indices are useful in distinguishing small-leaved species from other plant species.

##### 2.3.3.3 Labeling procedure

To train supervised learning algorithms and conduct image classification, a set of labeled ground truth data (with selected species classes) is needed. The sampling and labeling procedure was performed outside of the main processing chain using a polygon-based approach. This means that polygons were manually generated across the plots and then labeled by hand through visual image interpretation. The high spatial resolution UAV-derived RGB orthomosaic (pixel size of 2 mm) was used as a reference. The polygons were labeled as either grass (class 1), clover (class 2), plantain (class 3) or weeds (class 4). The polygon digitization was spatially randomized and concentrated on defining patches of species rather than single leaves. The 1167 labeled polygon were evenly spread across the analyzed plots and covered around 17% of the total plot area. Special attention was paid to the equal distribution of species classes among the polygons created. Nevertheless, plots were dominated by grass and clover with sporadic and scattered occurrences of plantain or weeds. Consequently, it was impossible to obtain an equal number of samples per class. In the end, grass was represented by 434, clover by 468, plantain by 192, and weeds by 73 labeled polygons.

These manually generated polygons which are treated as ground truth data (also known as reference data) were utilized to extract mean values from raster variables (spectral bands, CHMs, and vegetation indices), and were then used to build grouped boxplots and Principal Component Analysis (PCA). The main aim here was to identify and visualize the patterns within the dataset and understand the differences among classes and relationships among variables. Boxplots and PCAs were generated separately for data obtained with the RGB and with the multispectral sensor.

In this study, sample segments (learning database) used for OBIA classification were generated by applying spatial join of manually labeled polygons (ground truth data) and image segmentation results. The spatial join parameters were set as ‘within’ and ‘overlap’ to select segments that were entirely contained within the labeled polygons, as well as segments that partially overlapped the labeled polygons.

##### 2.3.3.4 Image classification

The prepared learning databases that can be used for the classification procedure were then exported to the RStudio (RStudio: IDE for R, R Studio Inc., Boston, MA, USA), where further analysis on model calibration and validation was conducted. In this study, we applied the Random Forest (RF) machine learning algorithm. Due to its high processing speed and great classification performance, RF has gained increasing attention over the past decades (Belgiu and Drăguţ, 2016). Several studies (Akcay et al., 2018; De Castro et al., 2018; Lu and He, 2018; Hall and Lara, 2022) have demonstrated that RF is suitable for land cover and species classification using high-resolution UAV imagery. The main reasons for selecting this classifier are its ability to manage large datasets with many variables (Akcay et al., 2018) and to adjust for interactions (correlations) among those predictor features (Hall and Lara, 2022). Random forest, introduced by Breiman (Breiman, 2001), is an ensemble learning technique that uses a combination of de-correlated decision trees. Such ensemble classifiers perform better, with higher accuracy and generalization capability than a single classifier (Rodriguez-Galiano et al., 2012).

Random Forest was trained for each prepared learning database (RGB and MS-based) using the *ranger* package (Wright and Ziegler, 2017) The following hyperparameters were considered: (1) the number of decision trees to be generated (*num.trees*) was set to default 500, as previous studies suggest that errors stabilize before this number of decision trees is reached (Belgiu and Drăguţ, 2016), (2) the number of variables selected and tested at each split (*mtry*) was set to the default square root of the number of input variables present in the learning dataset, and (3) minimal node size (*min.node.size*) was set to the default 1 for classification.

#### 2.3.4 Accuracy assessment

**Figure 4:**
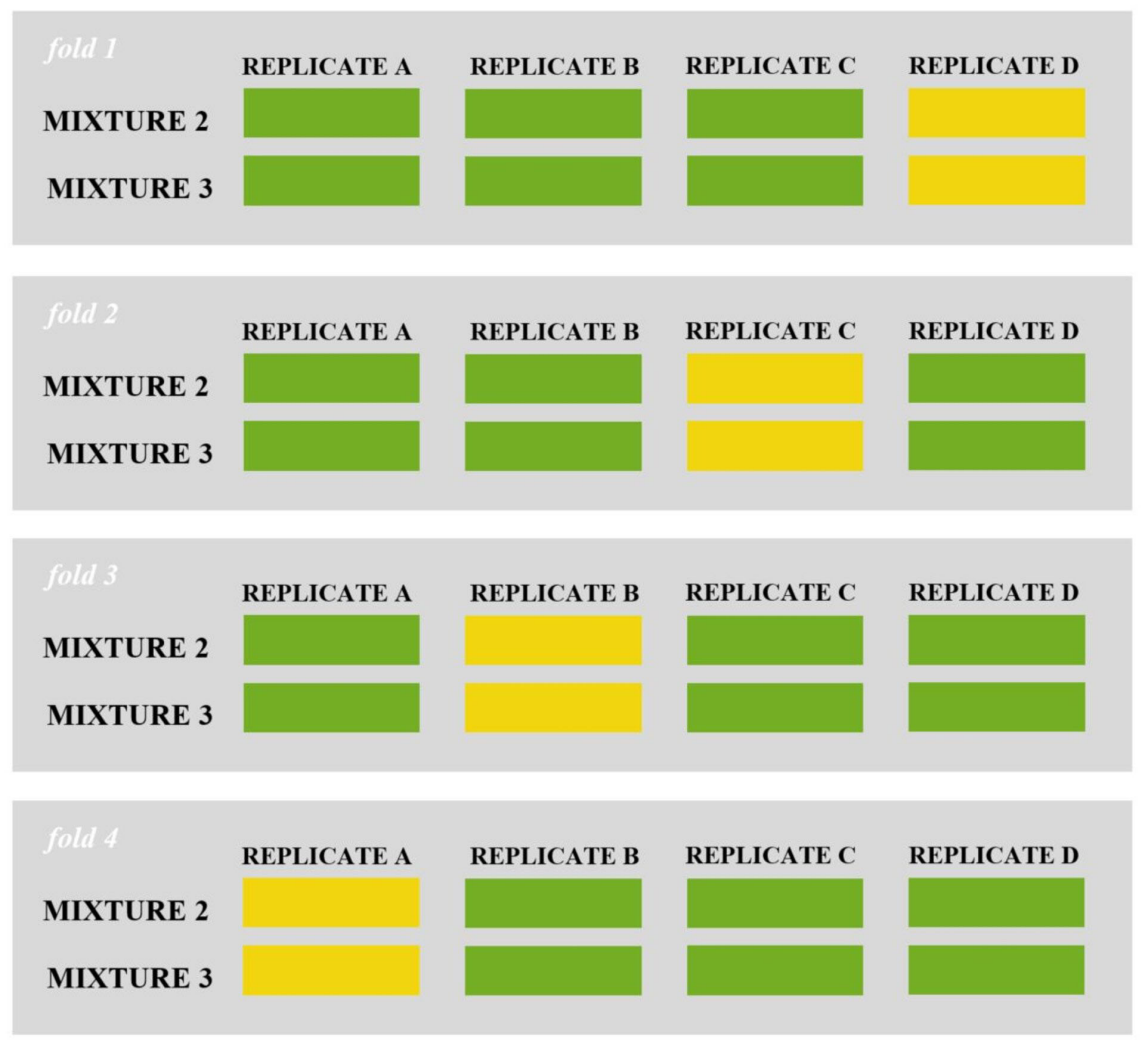
Visual representation of 4-fold cross-validation implemented in the study (green color: training set, orange color: test set).

As stated before (section 2.1.), only two types of species mixtures, totalling 8 plots, were chosen for Object-Based Image Analysis (OBIA): one comprised of grass and clover (mixture 2) and the other of grass, clover, and plantain (mixture 3). To assess the performance of the developed procedure, a form of spatial k-fold cross-validation approach was implemented. This involved resampling based on the location of observations, which was determined by the replicate scheme of the field trial. In total, four Random Forest (RF) classification models were built. Each model utilized 6 plots for training (from 3 different replicates and 2 mixtures), and the remaining 2 plots (from 1 replicate and 2 mixtures) were used to test the model. This process was repeated 4 times, using various replicate combinations, as illustrated in **Figure 4**.

Both qualitative evaluation through visual inspection and quantitative evaluation using reference data are integral components of the accuracy assessment in OBIA (Zhao et al., 2020). In supervised classification, a confusion matrix is a commonly used tool to organize information essential for accuracy assessment (Bratic et al., 2018). To summarize the confusion matrix information, several classification measures (scalar metrics) can be calculated. In this study, we focused on two metrics: a) the overall accuracy and b) the F1 score on a class-wise basis. The F1 score combines the harmonic mean of precision and recall (Guns et al., 2012), aiming to maximize both measures and obtain a better classifier. F1 evaluates model performance based on individual classes, as opposed to accuracy where the overall performance is computed. In general, an F1 score equal to 1 represents a model that perfectly classifies each observation into the correct class.

### 2.4 Species mapping

In order to obtain a classification map, a random forest (RF) model was built using labeled segments from all 8 plots. This model was then used to predict species class in all unclassified segments of the analyzed plots. The predicted class was used as a symbol label to create a species classification map. This procedure was repeated for different image segmentation results, obtained with different parameter combinations tested in the segmentation procedure.

## 3 Results

### 3.1 Multivariate relationships in reference data

**Figure 5:**
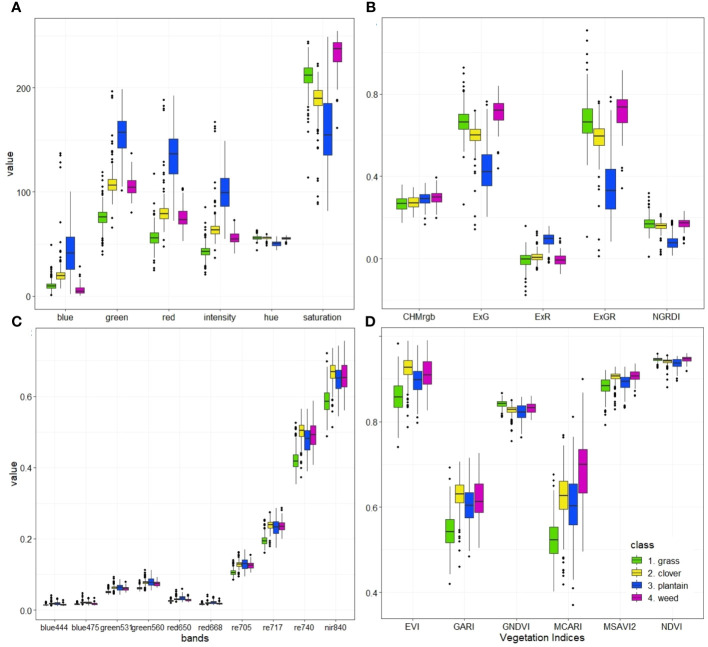
Visual summary (boxplots) of data extracted using labeled polygons as ground truth data for RGB-based color space with HIS color space **(A)** and RGB-based vegetation indices with CHM **(B)** MS-based spectral bands **(C)** and MS-based vegetation indices **(D)**.

**Figure 6:**
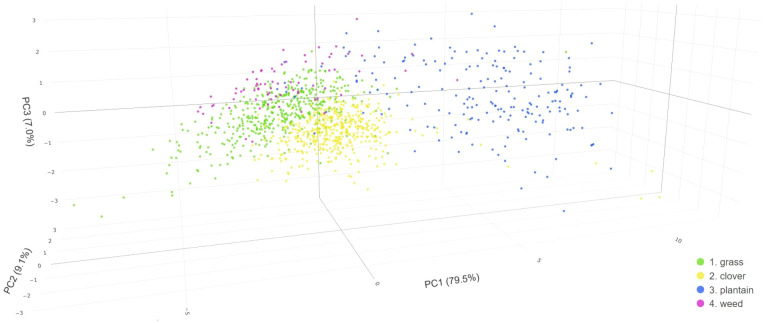
Principal Component Analysis (PCA) score plot representing RGB-based data of four distinct classes (grass, clover, plantain, and weed) obtained from labeled ground truth polygons, utilizing three dimensions.

**Figure 7:**
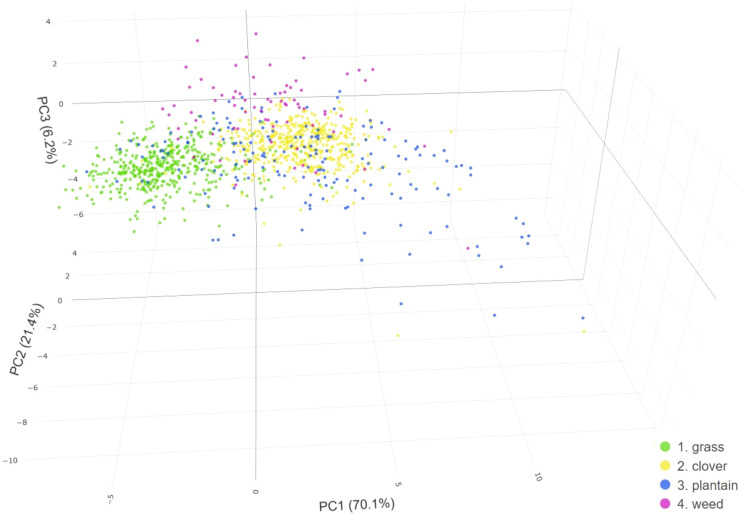
Principal Component Analysis (PCA) score plot representing MS-based data of four distinct classes (grass, clover, plantain, and weed) obtained from labeled ground truth polygons, utilizing three dimensions.

Mean values extracted from rasters (bands, CHMs and VIs) with manually generated polygons (ground truth data) were used to build grouped boxplots (**Figure 5**) and PCAs (**Figures 6**, **7**). The multivariate analysis indicates that spectral separation among the grass, clover, plantain, and weed classes is clearer with RGB imagery than with MS. What stands out in this figure is the difference between median and spread values of the plantain class for the RGB-derived data (**Figures 5A, B**). For almost all analyzed variables (except CHM, ExR, and NGRDI) the range of the plantain class is the largest, indicating wider distribution and more scattered data (spectral response). For MS-based data (**Figures 5C, D**) measures of spread and central tendency for plantain are closer to that of clover and weed class.

The interquartile range (the box heights) does not overlap between the grass and clover classes for the majority of analyzed variables (**Figure 5**). This indicates a clear difference between the two classes in terms of spectral response. Features, such as hue, CHM, ExR, ExGR and NGRDI, NDVI are an exception, as the median scores are similar for grass and clover and boxes overlap. On average, grass has lower values for almost all spectral bands and vegetation indices. On the contrary, when considering saturation, ExG, ExGR, and GNDVI features, grass shows higher values than clover. There are several similarities between clover and weed in their spectral response, as the range and median values are close to each other. Nonetheless, differences between these two classes can be observed in the RGB-based blue, ExG, and ExGR variables, as they are more aligned with the grass class. Canopy height has similar median and interquartile range values for the four analyzed classes.

The RGB-based 3D PCA plot presented in **Figure 6** shows that the first three principal components (PC1, PC2 & PC3) capture almost 96% of variance from the data. The first component (PC1) explains 79.5% of that variation, the second component (PC2) 9.1% and the third component (PC3) 7% of that variation. Hence, they are included in the further description and results.

The clusters in **Figure 6** reveal the presence of multiple distinct distributions within the data. There is not much overlap between plantain and the other three classes. The spread of observations is the largest for plantain, especially across PC1 and PC3. These samples are not as closely grouped as the grass or clover classes. There are a number of similarities and differences between grass and clover across the PCs. Both grass and clover samples share a similar spread of values across PC2. There is also a considerable overlap between these two classes along PC1 and PC3 However, when looking at observations in the three-dimensional space, one can see that grass and clover cluster separately. Even though both grass and clover observations cluster rather diagonally from low (negative) values on PC1 and PC3 space to higher values on PC1 and PC3, grass displays lower values on PC1 and higher values on PC3 than clover samples. The weed class overlaps to a large extent with the grass and clover classes in PC1 and PC2 values, but it differs when considering PC3. The weed class reaches higher values (on average) along PC3.

The next important step is to look into the relationship of the principal components with their original variables. For this purpose, the magnitude and the direction of linear coefficients, also known as loadings, were explored (**Supplementary Table S1**). The first principal component is primarily an equally weighted contrast between blue, green, intensity, red, and ExR variables (positive coefficients) and hue, saturation, ExG, ExGR, and NGRDI variables (negative coefficients). In contrast, the second principal component has a large positive association with CHM and very small contributions from all the other variables. The third principal component has the strongest weighted contrast between hue and saturation. Blue, NGRDI (negative coefficients), and ExR, CHM (positive coefficients) are other variables influencing PC3.

The 3D PCA plot on multispectral rasters (**Figure 7**), shows that nearly 98% of variance from the data is explained by the first three principal components (PC1, PC2, & PC3). PC1 captures 70.1% of the variability, followed by PC2 with 21.4%, and PC3 with 6.2%. Consequently, these components are considered for further description and analysis.

Similar to the RGB-based PCA, the plantain class displays the widest spread, particularly across PC1. However, in contrast to the RGB-based PCA, observations of the plantain category overlap with those of the other three classes. Also, in this case, grass and clover classes share several similarities and differences across the PCs. Observations of both classes have a similar spread of values across PC2 and PC3, but a different spread across PC1. While grass observations cluster around negative PC1 values, clover observations center around the positive PC1 values. Similar to the RGB-based PCA, the weed class intersects with other classes in PC1 and PC2 space, yet it diverges in PC3 dimension, where it typically reaches higher values.

The first principal component represents an evenly weighted sum of all analyzed variables (**Supplementary Table S2**). This PC has negative associations with GNDVI, Clg, SR, and NDVI, while it has positive associations with the remaining variables. Conversely, the second principal component shows the strongest positive correlation with MSAVI. Features such as EVI, NDVI, GARI, GNDVI or nir spectral band are the other variables influencing PC2. PC3 demonstrates the strongest weighted contrast between MCARI (positive coefficient) and SR with blue spectral band (negative coefficients).

### 3.2 Object-based image analysis: segmentation and classification results with accuracy assessment

**Figure 8:**
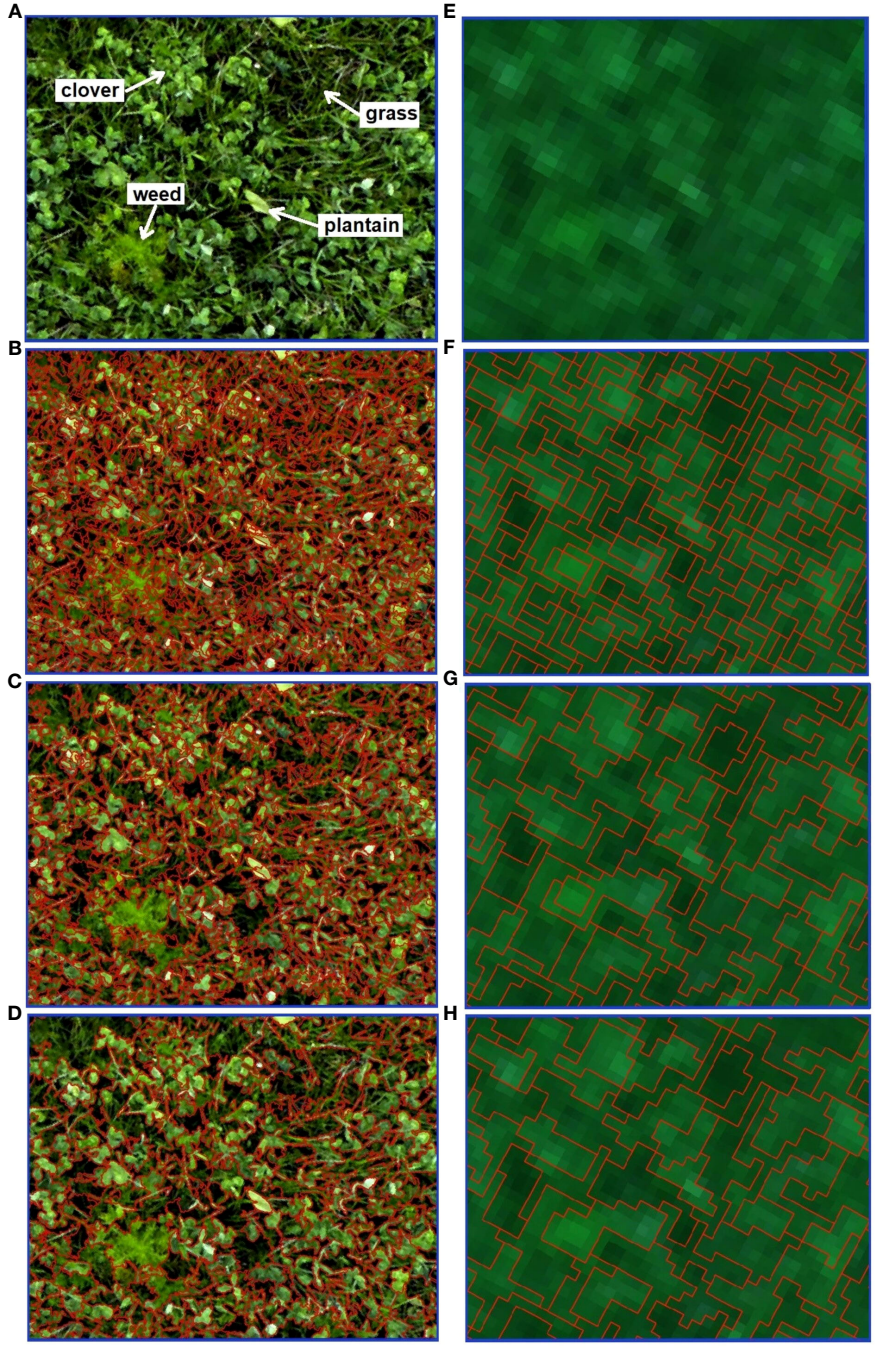
Image segmentation results of a B3 plot fragment, performed separately for RGB (left) and MS (right) imagery. RGB-based orthomosaic **(A)** and MS-based true color composite **(E)** are used as a background. The parameters of *minsize, ranger, spatialr* were set respectively, as follows: **(B)** 30, 10, 10; **(C)** 200, 10, 10; **(B)** 120, 30, 10; **(F)** 2, 0.01, 5; **(G)** 8, 0.02, 5; **(H)** 14, 0.01, 15.

In this study, different combinations of parameters were investigated and their impact on segmentation was explored and visualized. **Figure 8** illustrates the results of such image segmentation (red polygons) for both RGB (left side) and multispectral (right side) rasters. A few sets of segmentation parameters (*minsize, ranger*, and *spatialr*) were selected as an example to present varying levels of detail, with decreasing number of acquired segments (from top to bottom). **Figure 8A** also indicates grass, clover, plantain and weed classes, showcasing the variations.

As expected, the spatial resolution associated with the UAV sensor (RGB vs. MS) and its flight altitude, plays an essential role in defining the outcomes of the applied OBIA technique. With high spatial resolution imagery, like the one acquired with the RGB sensor, segmentation is possible with a very high level of detail. With lower resolution rasters (i.e. multispectral ones) we achieve coarser image segmentation. As shown in **Figure 8B**, pixels are clustered into very small objects such as individual grass leaves or clover leaflets. What is interesting about this segmentation outcome is that the single plantain leaf, as well as the weed plant (accentuated in **Figure 8A**), are separated into several objects. This is a consequence of over-segmentation, with multiple objects that represent a single feature. In the following segmentation (**Figure 8C**), the number of segments obtained was much lower. Certain plants are still depicted by more than one object (i.e. plantain leaf). While other plants, e.g. weeds or some grass patches are more homogeneous and spatially continuous. In this case, a weed plant is well-delineated into one object. The segmentation result shown in **Figure 8D** also shows a low number of segments and the boundaries between features are rather well-defined. For instance, in the top left corner of **Figure 8D**, a grass patch (comprising multiple grass blades) is clustered into one object, similarly to a clover patch located nearby.

Upon close examination of MS-based segmentation results (presented in **Figures 8F–H**), several notable differences with the RGB-based results can be observed. While MS imagery may lack the level of detail of RGB images, it is still able to distinguish patches of grass and clover, with grass appearing darker than clover. In MS rasters, plantain leaves do not stand out anymore and cannot be distinguished easily (mixed pixel problem, as pixels contain information from multiple ground cover classes). In addition, the weed plant closely resembles a clover patch. **Figure 8F** displays the most detailed MS-based segmentation, capturing the smallest objects. In the segmentation shown in **Figure 8G**), less detailed results are obtained with a reduced number of objects. The segmentation output shown in **Figure 8H** is a clear case of under-segmentation. Multiple features, representing different ground cover classes, are contained within a single segment.

**Figure 9:**
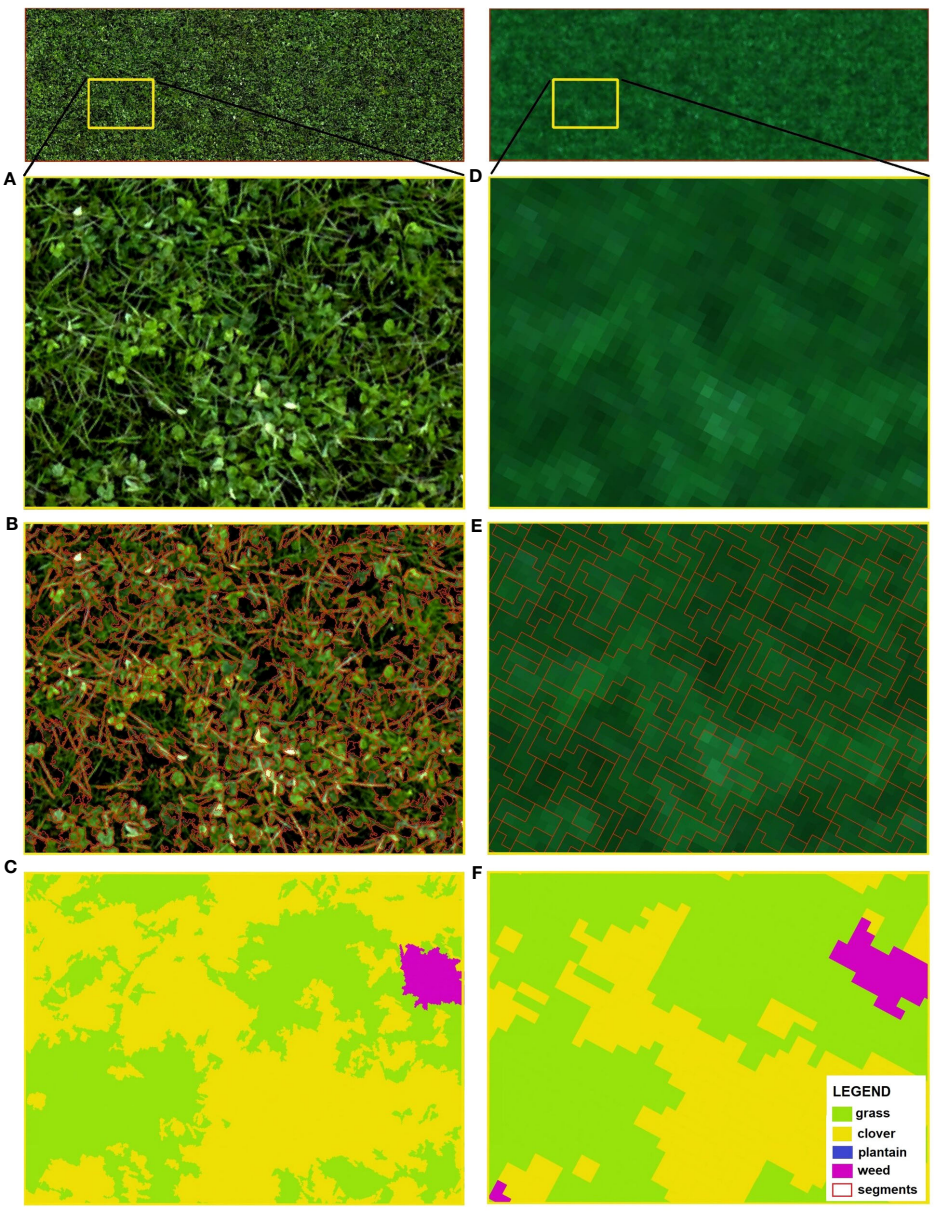
A C2 plot with a sown mixture of grass and clover species, the area highlighted by the yellow frame represents an area of interest (AOI) for further analysis (top). Enlarged view of AOI showing RGB-based orthomosaic **(A)** and MS-based true color composite **(D)** as input imagery for OBIA; results of image segmentation **(B, E)**; classification maps acquired with OBIA technique **(C, F)**, where green: grass, yellow: clover, blue: plantain, pink: weed.

**Figure 9** showcases the outcomes of species classification using UAV imagery with different spatial resolutions derived from both sensors. The C2 plot, sown with a mixture of grass-clover, and a blow-up of the yellow-framed area are used as examples of further analysis. The detailed RGB-based orthomosaic shown in **Figure 9A** and the MS-based true color composite shown in **Figure 9D** were utilized as input. Image segmentation using the mean-shift algorithm is shown in **Figures 9B, E**. Classification maps differentiating four classes (grass, clover, plantain, and weed) are presented in **Figures 9C, F**. The RGB-based classification yields a more intricate and detailed species map **Figure 9C** than the MS-based one (**Figure 9F**) But, as can be seen from the selected area of interest, both OBIA classification maps share some key similarities. The largest patches of clover and grass are identified in similar/corresponding areas. However, clover covers a larger area in the RGB-based classification map compared to the MS counterpart. The main difference is visible in the upper section, where a larger grass patch is predicted with coarser MS imagery. In both cases, a weed plant located on the upper right side (weed class) was correctly classified. However, the predicted coverage area of that weed is larger than in reality, particularly evident in the MS-based classification map. For instance, in **Figure 9F**, the small clover patch (bottom left) was partially misclassified as weeds. Visual inspection of the generated classification maps reveals that the OBIA procedure facilitates fine segmentation and classification of the UAV-derived imagery. It is possible to successfully identify the two primary cover classes (grass and clover), but distinguishing the remaining two classes (plantain and weeds) remains challenging.

**Figure 10:**
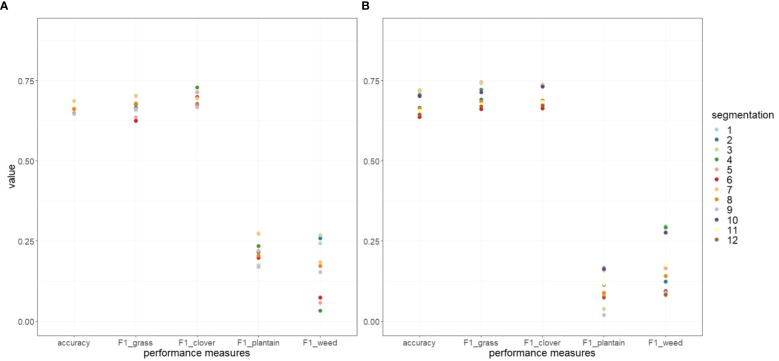
Performance assessment of multiresolution image segmentation using RGB **(A)** and multispectral **(B)** imagery, with different parameter combinations using Random Forest (RF) classifier and spatial k-fold cross-validation. The evaluation metrics included the overall accuracy and the F1 score on a class-wise basis (x-axis), having a range between 0 and 1 (y-axis).

The results of the performance assessment for OBIA are presented separately for RGB (**Figure 10A**) and MS (**Figure 10B**) imagery. The overall accuracy and the F1 score on a class-wise basis are the two metrics used for this evaluation (x-axis), with a range between 0 and 1 (y-axis). Outcomes for different segmentation parameter combinations used in the classification procedure (numbered from 1 to 12, presented in **Table 1**) are summarized. Segmentations 10, 11, and 12 from the RGB-based analysis are removed from further analysis due to insufficient object separation. The results were obtained by applying a Random Forest (RF) classifier and 4-fold cross-validation.

Both RGB and MS-based imagery yield good to moderate performance, with overall accuracies of approximately 70%. For both RGB and MS-based classification, the highest overall accuracy was achieved with segmentation 1, 4, and 7, with approximate accuracies of 0.69 and 0.72, respectively. On the contrary, the least accurate outcomes were recorded for segmentations 3, 6, 9 or 12, with accuracies of 0.65 and 0.64 for RGB and MS-based classification, respectively. Overall, segmentations that generated smaller objects demonstrated higher overall accuracy. This trend can be observed for both RGB and MS-based OBIA. The minsize parameter plays a key role in determining the size of segments and has thus the strongest influence on the achieved accuracy.

The highest F1 score for the clover class in RGB-based OBIA was achieved with segmentation 4 (F1 = 0.73), while in MS-based OBIA, segmentations 1 and 7 resulted in the highest F1 score (F1 = 0.74). Conversely, the lowest F1 score values of 0.67 (segmentations 3 and 9) for RGB and 0.66 (segmentations 3 and 6) for MS-based OBIA were noted. Similar F1 values were observed for the analyzed grass class. With high-resolution RGB imagery, the F1 score peaked at 0.7 (for segmentations 1 and 7) and was the lowest for segmentation 6 (0.62). With MS imagery, the F1 score was the highest for segmentation 1 (0.75) and the lowest for segmentations 3 and 6 (0.66). These results demonstrate that both RGB and MS-based OBIA yield similar F1 scores for the grass and clover class. The plantain and weed classes are particularly difficult to detect and yielded very low F1 scores. When compared, MS-based OBIA results in lower F1 values (on average) for the plantain class than for the weed class. The variability (spread) of F1 scores across different segmentation parameters is also more pronounced for the plantain and weed classes.

### 3.3 Clover fraction estimation

**Figure 11:**
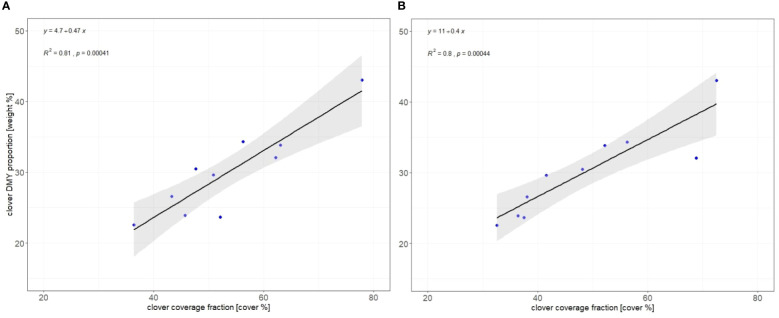
Relationship between the predicted clover cover (x-axis) and the harvested dry matter (y-axis) using linear regression with 95% confidence interval (grey area), regression line equation, Pearson correlation coefficient (R^2^ value), and p-value. Selected classification results, based on segmentation 4 for RGB **(A)** and segmentation 1 for MS **(B)** imagery, are given as an example.


To check the effectiveness of the OBIA procedure, we opted to explore the relationship between the OBIA results and the relative proportion of dry matter yield. Classification maps obtained with the OBIA technique were clipped by 10 subplots selected in the procedure (shown in **Figure 1**) and the cover area of each class was calculated. This information was coupled with the Dry Matter Yield (DMY) data collected in the field. The correlation between classification results (clover coverage) and corresponding dry matter proportion is presented in **Figure 11** by employing the following metrics, defined in Equations 1 and 2:

(1) clover coverage fraction [cover %]=clover coverage/total subplot area

(2) clover DMY proportion [weight %] = DMY of clover/total subplot DMY

A positive and strong relationship was found between the predicted clover cover and the dry matter fraction. Despite the lower resolution of the MS sensor imagery, similar correlations were observed between the clover ratio and the DMY proportion (0.81 and 0.8 for RGB and MS, respectively).

## 4 Discussion

Image analysis of mixed sward canopies faces numerous challenges due to the varying optical plant properties and varying leaf shapes and colors of the constituent species, as well as overlapping plant parts (Himstedt et al., 2009). Very high-resolution imagery can pose further challenges in species classification as it captures detailed features, such as gaps, shadows or nonuniform soil background, which may introduce noise and result in misclassification (Lu and He, 2018). Object-based image analysis (OBIA), as an alternative to pixel-based classification, segments an image into meaningful objects and assigns a specific vegetation class to each object (Blaschke et al., 2014). A recent study by Ventura et al. (2022) utilized OBIA classification and UAV imagery to monitor and map semi-natural grasslands. They successfully differentiated three grassland types, such as closed and open grasslands, achieving an overall classification accuracy of over 89%. Another study conducted by Lu and He (2017) employed OBIA on UAV-acquired images to investigate species composition in a tall grassland, focusing on brome, goldenrod, milkweed, and fescue species. They recorded an overall accuracy of approximately 85% across images obtained at different times.

The assessment of the accuracy of the OBIA approach applied in this study demonstrated good performance, achieving an overall accuracy of approximately 70%, for both RGB and MS-based imagery. The F1-score used to estimate the accuracy achieved for the four analyzed classes, offered a deeper insight into model performance. Grass and clover classes yielded similar F1 scores, exceeding values above 0.7 in both RGB and MS-based OBIA, which is an indication of good performance (Van Otten, 2023). It implies that roughly 70% of the samples were classified correctly. The confusion matrices show that clover was primarily misclassified as grass and that grass was predominantly misclassified as clover. Misclassification of clover and grass into other classes (plantain and weeds) was negligible. This pattern is consistent across RGB and MS imagery. In contrast, detecting the plantain and weed classes were challenging, with F1 scores reaching at best 0.27. According to the confusion matrix, both plantain and weeds were predominantly misclassified as grass and clover. In RGB-based classification, the misclassification of plantains and weeds was evenly distributed between the grass and clover classes, while in MS-based classification, plantain and weeds were more frequently misclassified as clover, probably because they are interpreted as a dicot due to the lower resolution images. Furthermore, lower performance (lower F1 scores, depicted in **Figure 10**) for the plantain and weed classes was to be expected due to imbalances in the dataset: grass and clover classes had considerably more instances (labeled polygons) than the other two classes. Consequently, classifiers tend to overlook the less represented classes while focusing on and prioritizing classes with higher representation (Lin and Chen, 2013) in the learning database.

Similarities in the spectral response among the classes can also be a reason for misclassification, as explained in section 3.1. In MS data, the measures of spread and central tendency for plantains are closer to those of clover and weed class. This could be explained by the lower spatial resolution of MS rasters, along with the small size of the plantain polygons used for data extraction. Here, a mixed pixel problem arises, as other classes contribute to the spectral response of plantains. In consequence, delineating segments containing plantains proved to be challenging. Additionally, similarities in multispectral response between weed and clover classes could possibly explain difficulties in the delineation and classification of weed segments.

To examine the effectiveness of the OBIA procedure, the relationship between the predicted clover coverage and the relative proportion of harvestable dry matter yield was investigated. A positive and strong relationship was found between the predicted clover fraction and DMY proportion, with R^2^ values exceeding 0.8 for RGB and MS-based OBIA classification results. Remarkably, even though the MS imagery has a lower resolution, comparable correlations were found, underscoring the applicability of the analysis across different data resolutions. Nevertheless, as a relatively small sample size (10 subplots) was used to examine the relationship between the obtained clover cover and DMY proportion, these findings should be interpreted with caution. Further research (with additional data collection) is necessary for deeper investigation of these relationships. Image segmentation stands as the initial step in OBIA, and it partitions the entire image into distinct, non-overlapping segments (Marçal and Rodrigues, 2009), with meaningful representation. Hence, an optimal segmentation should yield an image partitioning where each segment corresponds (as much as possible) to an object of interest in the study area (Troya-Galvis et al., 2015), while still maintaining high internal within-segment homogeneity (Hao et al., 2021). This makes the segmentation parameter selection a critical step in the OBIA procedure. Segmentation errors can be due either to over-segmentation or to under-segmentation. Over-segmentation occurs when a single object of interest is segmented into too many polygons, whereas under-segmentation happens when multiple objects of interest are included in a single segment (Troya-Galvis et al., 2015; Hao et al., 2021). As shown in **Figure 10**, segmentations which generated more and smaller segments (e.g. 1, 4 or 7) resulted in higher overall accuracy and F1 scores for the grass and clover class. A similar trend was observed with clover fraction estimation outcomes. In general, the correlations between clover coverage (classification results) and corresponding dry matter yield proportion achieved better R^2^ values when more detailed segmentation was obtained in the OBIA procedure.

The *minsize* parameter also referred to as the scale parameter, which determines the relative segment size (Drǎguţ et al., 2010), has a substantial impact on OBIA and classification results (Zhao et al., 2020; Hao et al., 2021). Possibly, setting a smaller *minsize* parameter reduces the probability of segmenting multiple classes into a single object, leading to increased overall accuracy. These findings are consistent with Huang et al. (2020) who showed that a smaller scale parameter produces higher accuracy. Correspondingly, Grippa et al. (2017) advocated for over-segmentation over under-segmentation, arguing that the former allows for correction during the classification phase. The findings of this study corroborate these conclusions. Therefore, employing segmentations that generate numerous smaller segments should be preferred. Nevertheless, performing detailed segmentation, particularly with high-spatial resolution RGB imagery is time-consuming and computationally intensive. Therefore, a selection of technology (sensors, segmentation parameters) would depend on the end users and their objectives (e.g., acceptable error margins or required decision-making time).

**Figure 12:**
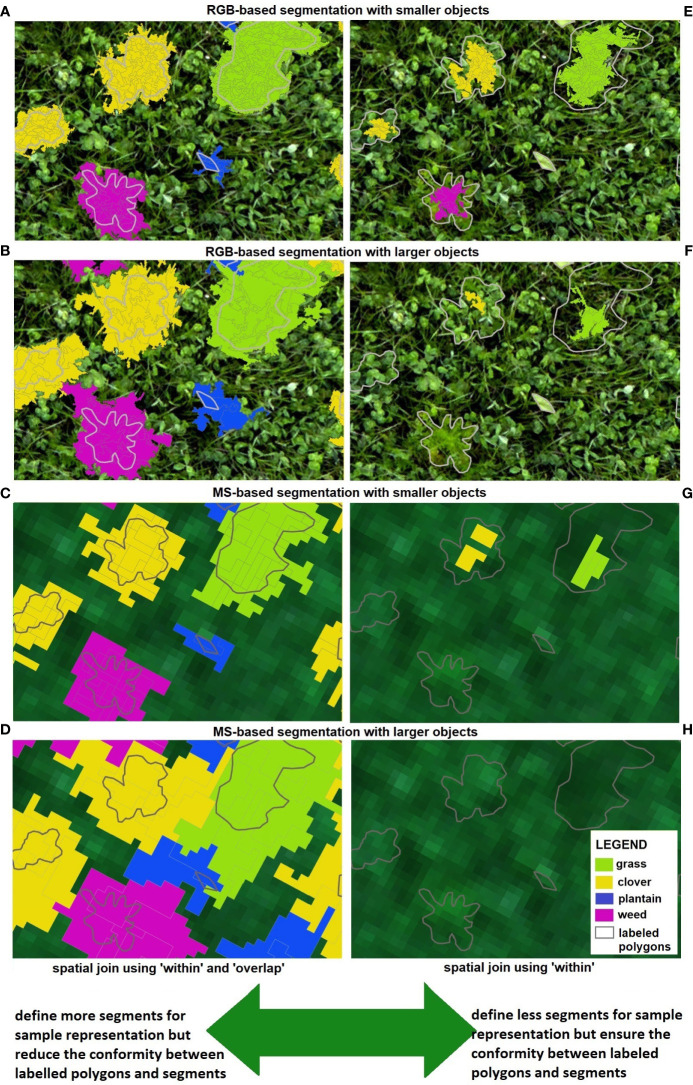
Defining sample segments (learning database) for OBIA classification. In this study, ‘within’ and ‘overlap’ parameters were selected when applying the spatial join tool, presented on the left side **(A–D)**. For a comparison, only the ‘within’ parameter could be selected, shown on the right side **(E–H)**. Examples of different segmentation results are shown here, with RGB-based segmentation creating smaller **(A, E)** and larger objects **(B, F)**; and MS-based segmentation creating smaller **(C, G)** and larger objects **(D, H)**.

In this study, sample segments used for OBIA classification assessment were generated with spatial join where ‘within’ and ‘overlap’ parameters were selected. As a result, a higher number of segments were selected for sample representation which led to decreased agreement between samples and segments, treated as a limitation of the selected method. While this approach performs better with segmentation that generates smaller objects (e.g., **Figure 12A** for RGB-based OBIA and **Figure 12C** for MS-based OBIA), it shows limitations with larger segments (e.g., **Figures 12B, D**). With such larger objects, there is a tendency to incorporate segments into the sample representation that, in reality, belong to a different class. The evidence of this can be seen in the center of graphs B and D where segments representing grass and clover were incorrectly assigned to the plantain class. Larger objects also present a higher likelihood of assigning multiple classes into a single segment. This can be seen in the bottom of graphs B & D, where one bigger segment that actually represents both a weed plant and a patch of clovers was assigned as a weed class.

An alternative approach that would enhance conformity between labeled polygons and segments is to define fewer segments for sample representation. This option is depicted in **Figure 12** (right side), where only the ‘within’ parameter was applied. This means that only segments whose entire geometry is enclosed by the labeled polygon, without any shared boundary points, are considered. While this approach might be effective for RGB-based OBIA, particularly for segmentation that generates smaller objects (**Figure 12E**). It is not suitable for MS-based OBIA, especially when dealing with segmentation that produces larger objects (like in **Figure 12H**), where no segments would be selected to represent the analyzed classes and build a learning database. This issue can be further illustrated in graphs F and G, where only a few segments were chosen for the grass and clover classes, but none were selected for the weed and plantain classes. It seems that the results presented in **Figure 10** (F1 scores) could be improved if the selection of sample segments is refined, by minimizing incorrect assignment of segments to a class. This improvement could then be utilized to construct an enhanced learning database for OBIA classification.

When extensive fields need to be assessed, visual inspection of clover coverage and proportion becomes impractical (Skovsen et al., 2017). Hence, automated and efficient methods are deemed necessary. Farmers and breeders could greatly benefit from tools, that enable visualization of clover spatial distribution, like the ones examined in this study. The main question here revolves around the end-user of such outcomes (e.g., classification maps). While very detailed segmentations and classifications may be useful for scientific analysis and applications, for a farmer, coarser maps showing the approximate distribution of grass-clovers patches across the field may suffice. With this objective in mind, it becomes apparent that high-resolution RGB imagery is not necessary and multispectral data with lower resolution and faster processing time can be successfully applied using the OBIA procedure.

The classification maps derived from both RGB and MS-based OBIA hold potential for agricultural applications. They could be leveraged by farmers for forage quality assessments and for optimizing agronomic tasks, such as the need for resowing or fine-tuning fertilizer applications in a precision agriculture context. While the determination of some of the analyzed classes (plantain and weeds) with MS-based OBIA is unsatisfactory, it could certainly be utilized to differentiate between monocots (grass) and dicots (clover, weeds, and plantain possibly grouped into one class). Even though the results for the plantain and weed classes obtained with RGB-based OBIA were disappointing, they still outperformed the MS-based OBIA. Several possible factors, such as clearer distinction of spectral responses among classes (**Figure 5**) and higher resolution of RGB camera (reducing mixed pixel problem), could explain this.

## 5 Conclusions

Given the significance of having automated and efficient methods for estimating legume coverage and proportion in mixed swards, we proposed an open-source OBIA approach. This study aimed to detect and quantify the ratio of species within mixed grass-clover swards, while also providing a simpler procedure suitable for breeders and farmers (as an alternative to more complex deep learning methods). A UAV was employed to capture high spatial resolution imagery with an RGB and a multispectral sensor, achieving 2 mm and 2 cm spatial resolution, respectively. The findings showed that both RGB and MS-based OBIA yielded good performances, where comparable F1 scores for the grass and clover classes were reached (exceeding 0.7 values). Conversely, identifying less prevalent plantain and weed classes posed challenges, resulting in low F1 scores. Both RGB and multispectral sensors yielded comparable results. However, the choice between them depends on the specific objectives of the application. For instance, when coarse maps delineating the distribution of grass-clover patches suffice, multispectral data with lower resolution and faster processing times may be preferred. The strong correlation observed between predicted clover fraction and dry matter yield proportion highlights the potential of the proposed procedure for estimating and visualizing clover coverage in mixed grass-clover fields. Such findings indicate the practical applicability in providing valuable support for breeders and also farmers operating within the realm of precision agriculture. Moving forward, further research could explore refinements of this methodology to enhance its utility across different sites and environmental conditions.

## Data availability statement

The raw data supporting the conclusions of this article will be made available by the authors, without undue reservation.

## Author contributions

JP: Conceptualization, Data curation, Formal analysis, Investigation, Methodology, Visualization, Writing – original draft, Writing – review & editing. IB: Conceptualization, Data curation, Investigation, Methodology, Writing – review & editing. PQ: Formal analysis, Methodology, Visualization, Writing – review & editing. TD: Conceptualization, Formal analysis, Investigation, Methodology, Writing – review & editing. TV: Data curation, Resources, Writing – review & editing. KW: Resources, Writing – review & editing. GR: Conceptualization, Funding acquisition, Writing – review & editing. IJ: Funding acquisition, Supervision, Writing – review & editing. IR: Conceptualization, Supervision, Writing – review & editing. PL: Conceptualization, Formal analysis, Funding acquisition, Investigation, Methodology, Supervision, Visualization, Writing – original draft, Writing – review & editing.
