# Supplementary material for: Quantification of species composition in grass-clover swards using RGB and multispectral UAV imagery and machine learning
Source: Front Plant Sci. 2024 Jun 19;15:1414181. doi: 10.3389/fpls.2024.1414181 (PMC11219903; doi:10.3389/fpls.2024.1414181)
Supplement: Supplementary file 1 [file Table_1.docx]

Supplementary Table S1. The matrix of variable loadings for the RGB-based principal component analysis.

| variable | PC1 | PC2 | PC3 |
| --- | --- | --- | --- |
| blue_mean | 0.308 | 0.030 | -0.391 |
| CHMrgb_mean | 0.078 | 0.940 | 0.228 |
| ExG_mean | -0.330 | 0.068 | 0.121 |
| ExGR_mean | -0.332 | 0.092 | -0.016 |
| ExR_mean | 0.317 | -0.144 | 0.340 |
| green_mean | 0.311 | 0.142 | -0.067 |
| hue_mean | -0.287 | 0.161 | -0.550 |
| intensity_mean | 0.324 | 0.106 | -0.190 |
| NGRDI_mean | -0.322 | 0.136 | -0.298 |
| red_mean | 0.326 | 0.079 | -0.002 |
| saturation_mean | -0.293 | -0.004 | 0.482 |

Supplementary Table S2. The matrix of variable loadings for the MS-based principal component analysis.

| variable | PC1 | PC2 | PC3 |
| --- | --- | --- | --- |
| blue444_mean | 0.254 | -0.102 | -0.319 |
| blue475_mean | 0.259 | -0.102 | -0.276 |
| CLg_mean | -0.225 | 0.266 | -0.221 |
| EVI_mean | 0.193 | 0.355 | -0.175 |
| GARI_mean | 0.219 | 0.309 | -0.146 |
| GNDVI_mean | -0.224 | 0.281 | -0.171 |
| green531_mean | 0.279 | -0.028 | 0.028 |
| green560_mean | 0.277 | -0.024 | 0.059 |
| MCARI_mean | 0.179 | 0.230 | 0.565 |
| MSAVI2_mean | 0.106 | 0.465 | -0.015 |
| NDVI_mean | -0.189 | 0.352 | 0.219 |
| nir840_mean | 0.230 | 0.283 | -0.127 |
| red650_mean | 0.261 | -0.147 | -0.110 |
| red668_mean | 0.255 | -0.156 | -0.248 |
| rededge705_mean | 0.277 | 0.008 | 0.111 |
| rededge717_mean | 0.272 | 0.096 | 0.133 |
| rededge740_mean | 0.253 | 0.212 | 0.020 |
| SR_mean | -0.218 | 0.181 | -0.447 |
